# Erythroderma combined with deeper dermal dermatophytosis due to *Trichophyton rubrum* in a patient with myasthenia gravis: first case report and literature review

**DOI:** 10.1186/s12879-023-08752-5

**Published:** 2023-11-13

**Authors:** Henan Si, Yang Li, Zhiyang Huang, Yan Cui, Shanshan Li

**Affiliations:** Department of Dermatology and Venerology, First Hospital of Jilin University, Changchun, Jilin 130021 China

**Keywords:** Erythroderma, Deeper dermal dermatophytosis, *Trichophyton rubrum*, Dermatophyte infections

## Abstract

**Background:**

Dermatophytes are the most common causative pathogens of mycoses worldwide and usually cause superficial infections. However, they can enter deep into the dermis lead to invasive dermatophytosis such as deeper dermal dermatophytosis on rare occasions. Erythroderma is a severe dermatological manifestation of various diseases resulting in generalized skin redness, but erythroderma due to fungi infections is barely reported. In this article, we reported the first case of erythroderma combined with deeper dermal dermatophytosis due to *Trichophyton rubrum* (*T. rubrum*) in a patient with myasthenia gravis.

**Case presentation:**

A 48-year-old man was hospitalized because of erythema with scaling and nodules covering his body for a month. The patient had a history of myasthenia gravis controlled by regularly taking prednisolone for > 10 years and accompanied by onychomycosis and tinea pedis lasting > 8 years. Based on histopathological examinations, fungal cultures, and DNA sequencing results, the patient was finally diagnosed with dermatophyte-induced erythroderma combined with deeper dermal dermatophytosis caused by *T. rubrum*. After 2 weeks of antifungal treatment, the patient had recovered well.

**Conclusions:**

This case report shows that immunosuppressed patients with long histories of superficial mycoses tend to have a higher risk of developing invasive dermatophytic infections or disseminated fungal infections. Dermatologists should be alert to this condition and promptly treat the superficial dermatophytosis.

## Background

Dermatophytes represent a group of specialized filamentous fungi that cause superficial cutaneous infections, mostly restricted to keratinized tissues, in humans and animals. Based on the new taxonomy in 2017 [1], this fungal group consists of more than 50 species distributed in the genera of *Trichophyton*, *Microsporum*, *Epidermophyton*, *Nannizzia*, *Arthroderma*, *Lophophyton* and *Paraphyton*. Anthropophilic species infecting exclusively humans can use keratin from skin, hair, and nails as nutrient sources by producing abundant enzymes [2]. In rare cases, dermatophytes invade the dermis, subcutaneous tissues or extracutaneous organs, causing invasive dermatophytosis in patients with gene deficiencies, or in immunocompromised patients such as those who have undergone solid organ transplantation, long-term immunosuppressive treatments, or human immunodeficiency virus (HIV) infection [3, 4]. According to Durdu et al. [5], invasive dermatophytosis can be classified into four forms: (i) Majocchi’s granuloma, (ii) deeper dermal dermatophytosis, (iii) mycetoma and pseudomycetoma and (iv) disseminated dermatophytosis. Deeper dermal dermatophytosis most commonly presents as erythematous papules or nodules subsequent to ulcers and erythematous plaques and often occurs as an isolated lesion. Multisite infections combined with generalized superficial cutaneous symptoms are extremely rare.

Erythroderma, also known as generalized exfoliative dermatitis or exfoliative erythroderma, is a severe inflammatory skin syndrome with erythema and desquamation involving more than 90% of the body surface area. The most common cause of erythroderma is psoriasis, followed by eczema, drug-induced reactions, pityriasis rubra pilaris, and cutaneous T-cell lymphomas [6]. Erythroderma due to microbial infections, especially fungi, is very rare.

Here, we report a case of erythroderma combined with deep dermatophytosis caused by *T. rubrum* in a 48-year-old man with myasthenia gravis lasting for 10 years, which to date, has never been reported. Moreover, we performed two literature reviews to further analyze the characteristics of patients diagnosed with dermatophyte-associated erythroderma or deeper dermal dermatophytosis caused by *T. rubrum*.

## Case presentation

A 48-year-old man was admitted to the hospital for erythema, scaling and nodules covering his body for 1 month, which had gradually worsened. One month prior, erythema developed on the patient’s trunk and limbs with occasional itching, which gradually expanded and affected the whole body. He received no regular treatment at that time, except for intermittent topical humectant, which had no satisfactory effect. The patient simultaneously developed exophytic nodules on his lower extremities, which gradually grew, involving the trunk, upper limbs and scalp. Three days prior to the consult, the patient developed fever, accompanied by coughing and sticky white sputum. His highest body temperature was 39℃.

**Fig. 1:**
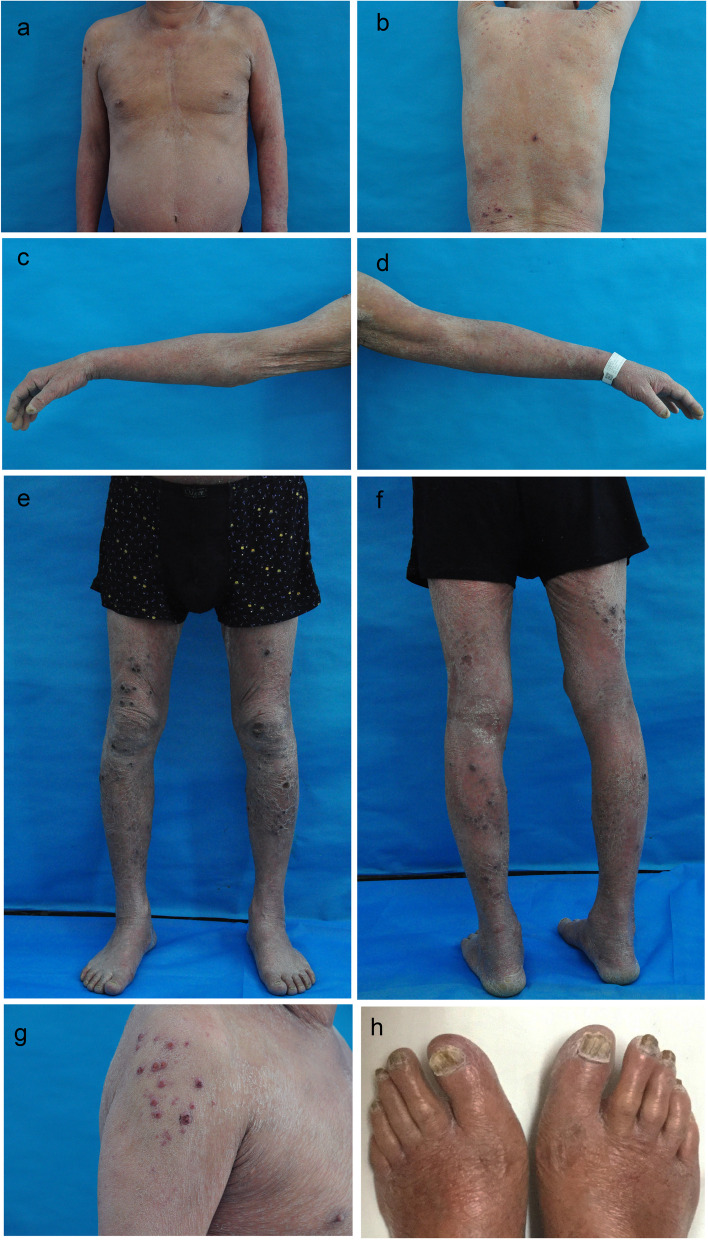
Clinical manifestations. **a**-**h** Erythema accompanied by scales covered the patient’s body, and multiple disseminated, firm and dusky red to purple nodules and plaques were distributed over his trunk (**a**, **b**), arms (**c**, **d**, **g**) and lower extremities (**e**, **f**), presenting with crusts and erosions. **h** All toenails were thickened, dystrophic and showed yellowish discoloration

Physical examination showed moon face, and dermatological examination revealed diffuse edematous erythema (affected area ≥ 90%) accompanied by scales covering his body. Multiple disseminated, firm and dusky red to purple nodules and plaques were distributed over his scalp, trunk, arms and lower extremities, with diameters of 1–2 cm, presenting with crusts and erosions. The fingernails and toenails were thickened, dystrophic and showed yellowish discoloration(Fig. 1a–h). The rest of the physical examination was unremarkable.

The patient had complicated previous histories, including chronic onychomycosis (8 years ago) and tinea pedis (13 years ago), which had occasionally been treated with topical antifungals, and myasthenia gravis, which was treated with 14–32 mg systemic prednisolone daily for 10 years after a thymectomy, along with 3 mg tacrolimus once daily for the preceding 2 months. He had a history of eczema for 5 years and was treated intermittently with topical hormones. His history also included treatment for adult-onset diabetes mellitus with 2-mg repaglinide tablets three times daily. The patient was preliminarily diagnosed with erythroderma and was hospitalized.

Serologic testing was performed on admission and showed total serum protein 52.9 g/L (normal 65.0–85.0 g/L), albumin 24.6 g/L (normal 40.0–55.0 g/L), IgE 3010.00 IU/mL (normal 100.0 IU/mL), blood glucose 13.56 mmol/L (normal 3.9–6.1 mmol/L), glycosylated hemoglobin 10.20% (normal 4.27%–6.07%), urine glucose 3+, erythrocyte sedimentation rate 37 mm/1 h (normal 0–15 mm/1 h), CD3+ T cell 89.20% (normal 54.02%–80.04%), CD19+ B cell 2.12% (normal 5.52%–19.47%), CD3-CD56+ NK cell 7.07% (normal 9.02%–34.57%), CD3+ CD56+ NK T cell 0.76% (normal 1.63%–16.87%), CD8+ CD38+ activated T cells 21.90% (normal 3.40%–18.21%), CD3+ T-cell count 557.6/µL (normal 723.5–1755.5/µL), CD8+ T-cell count 185.6/µL (normal 236.3–846.9/µL), CD19+ B cell count 13.3/µL (normal 86.6–388.1/µL), and CD3-CD56+ NK cell count 44.2/µL (normal 130.8–692.5/µL). Routine blood tests, kidney function, immunoglobulins, rheumatoid factor, and antinuclear antibody series were normal; HIV and rapid plasma reagin tests were negative. Chest computed tomography showed pneumonia. Sputum culturing revealed a moderate amount of *Candida albicans*.

**Fig. 2:**
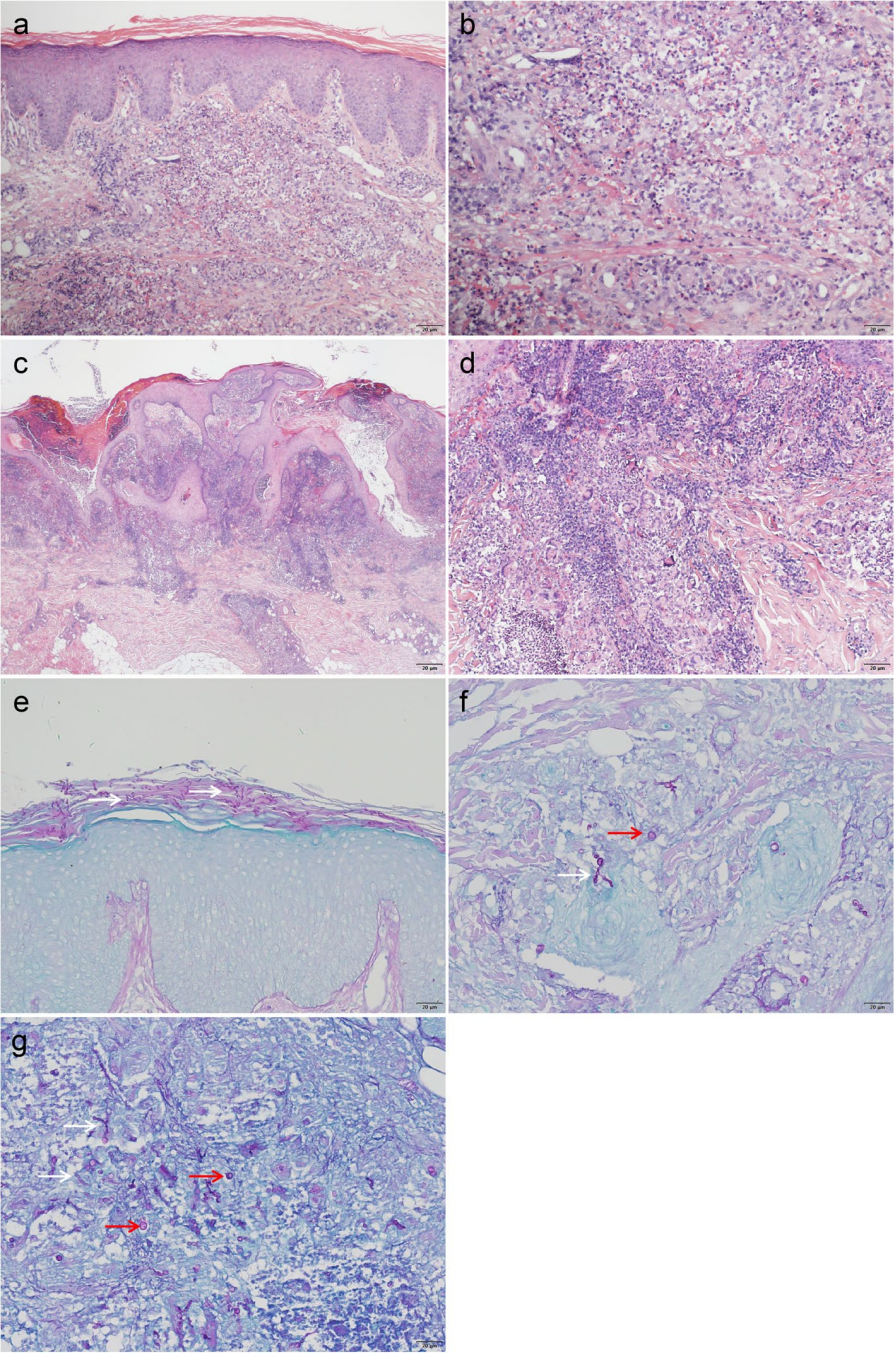
Histological description of the erythema and nodules. **a**, **b** Histological examination of the erythema revealed epidermal hyperplasia and hypertrophy, infectious granuloma in the dermis, and dense inflammatory infiltrate consisting of epithelioid cells, lymphocytes, plasma cells, neutrophils, and scattered multinucleated giant cells. Hematoxylin and eosin (HE) ×100 (**a**), HE ×200 (**b**). **c**, **d** Histopathology of the nodules revealed pseudoepitheliomatous epidermal hyperplasia, inflammatory granulomatous infiltration, and dense inflammatory infiltration in the dermis, with infiltrating cells identical to those of the erythema. HE, ×20 (**c**), HE, ×100 (**d**). **e**–**g** Hyphae (white arrow) and conidia (red arrow) in the corneum (**e**) and dermis (**f**, **g**). Periodic acid-Schiff stain, ×200.Scale bar, 20 µm

Two skin biopsies collected from different lesions (a nodule on the patient’s right upper arm and an erythema on his left forearm) were each divided into two parts; one was fixed, routinely processed, and stained with hematoxylin and eosin (HE) and periodic acid-Schiff (PAS) for routine histology; the other was used for fungal culturing. Histological examination of the erythema showed hyperplasia and hypertrophy of the epidermis, infectious granuloma of the dermis and dense inflammatory infiltrate, including epithelioid cells, lymphocytes, plasma cells, neutrophils and scattered multinucleated giant cells (Fig. 2a, b). Histopathology of the nodule showed pseudoepitheliomatous epidermal hyperplasia, inflammatory granulomatous infiltration and dense inflammatory infiltration in the dermis. The infiltrating cells were the same as those of the erythema (Fig. 2c, d). Immunohistochemical staining of erythema showed sporadic positivity for CD4, while CD3, CD8, and CD20 were all negative. PAS staining of both sections of the erythema and nodule revealed abundant thick septate hyphae and conidia, consistent with a fungal infection. The hyphae and conidia were found in both the stratum corneum and dermis of the erythema (Fig. 2e, f) but in only in the dermis of the nodule (Fig. 2g).

**Fig. 3:**
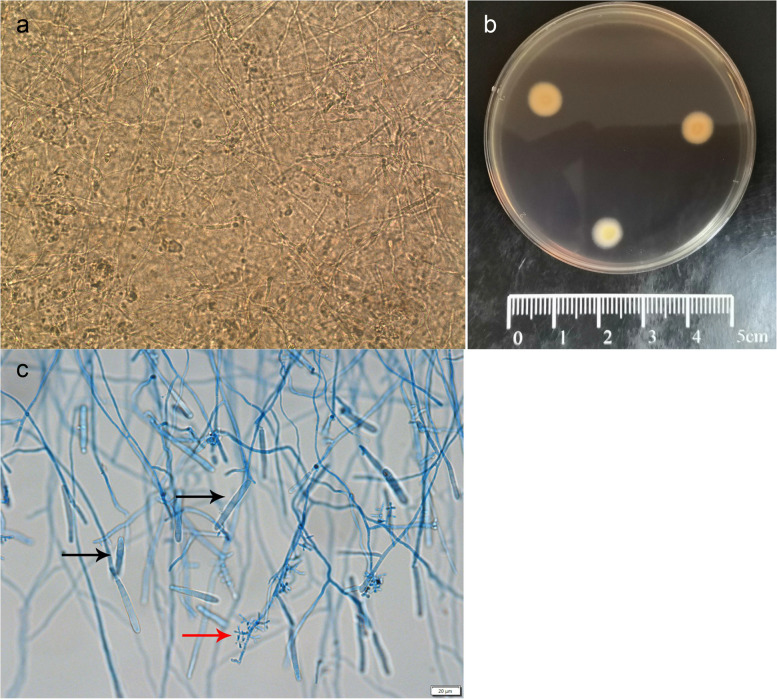
Fungal examination. **a** KOH wet mount and direct microscopic examination of the scrapings from the patient’s trunk showing abundant septate hyphae. ×200. **b** Small white fluffy and creamy yellow colonies were observed on potato dextrose agar plate after 1 week of culturing at 28 °C. **c** Macroconidia (black arrow) and microconidia (red arrow) were observed under lactate phenol medan staining with an optical microscope. Scale bar, 20 µm

Using sterile scalpel blades, scrapings from the trunk, foot and nail clippings were separated into two parts each. One was fixed with 10% KOH for direct microscopic examination, which showed fungal hyphae (Fig. 3a); the other was used for fungal culturing, for which the scrapings and skin biopsy samples were collected as described above, inoculated directly onto Sabouraud glucose agar slants (BD Difco,Sparks, MD, USA) and incubated at 28℃ for 1 week. All cultures showed single colonies, which were subcultured on potato dextrose agar plates (BD Difco, Sparks, MD, USA) for 1 week. The cultures yielded numerous small, white, fluffy (obverse) and creamy yellow (reverse) fungal colonies (Fig. 3b). The isolates were numbered as FHJU 19100101 (from the erythema) and FHJU 19100102 (from the nodule). Microscopic examination revealed both macroconidia and microconidia (Fig. 3c). Morphologic and microscopic characteristics suggested dermatophytes and were confirmed as *Trichophyton rubrum* via PCR and sequencing based on the internal transcribed spacer region gene (GenBank accession numbers: OM899647 for FHJU19100101 and OM899680 for FHJU19100102).

**Fig. 4:**
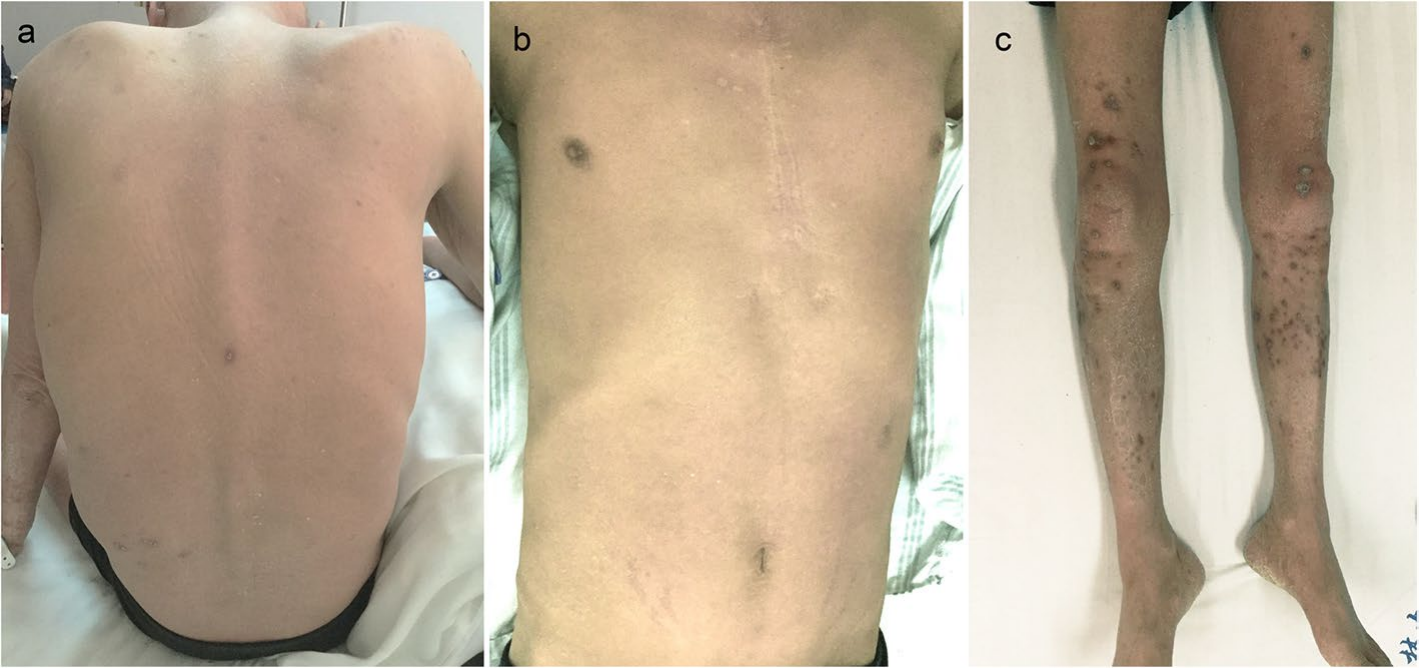
Clinical manifestations of the patient after treatment. **a**–**c** After receiving antifungal treatment for 14 days, the patient’s erythema and scales partially subsided

Based on these findings, the patient was diagnosed with dermatophyte-induced erythroderma and deeper dermal dermatophytosis. In addition to treatment for myasthenia gravis and diabetes, he was treated with 200 mg oral itraconazole twice daily and topical bifonazole cream once daily because direct examination (10% KOH) of the scrapings from the erythema and nail clippings showed fungal hyphae. Sputum culturing was performed because the patient exhibited coughing and expectoration. The patient’s temperature returned to normal after 2 days of treatment. Four days later, when a moderate amount of *Candida albicans* was reported from the sputum culture, and PAS staining of the biopsy revealed hyphae and spores, the treatment was switched to 0.2 g intravenous voriconazole twice daily plus 250 mg oral terbinafine once daily. After 14 days of treatment, the sputum culture yielded no fungal growth, and the erythema partially subsided (Fig. 4a–c). The patient was then discharged and prescribed 200 mg itraconazole twice daily as his condition improved. One month later during a follow-up, it was observed that the erythema had significantly diminished, and the nodules had improved. Unfortunately, the patient passed away due to another illness 2 months later.

## Discussion and conclusions

*Trichophyton rubrum* is the most prevalent species in human superficial mycoses, accounting for approximately 69.5% of all dermatophyte-associated infections [7]. *T. rubrum* usually causes superficial dermal infections that are limited to keratinized tissues (skin, hair and nails) [8]. Various elements of the innate immune system, including neutrophils, macrophages and mast cells, limit the infection to the epidermis. The keratinized layer and normal flora of the skin act as physical barriers preventing deeper invasion. Dermatophytes also invade the keratinized tissues and cause dermatophytosis in individuals with innate or acquired immunodeficiencies such as solid organ transplants, HIV infection, and gene deficiency [9].

Myasthenia gravis is a typical autoimmune disease mediated by autoantibodies against the nicotinic receptor of acetylcholine in neuromuscular junctions and involves fatigable skeletal muscle weakness [10]. Pyridostigmine and corticosteroids play traditionally central roles in managing myasthenia gravis. Thymectomy is recommended for patients with thymoma with myasthenia gravis [11]. These management strategies, especially long-term use of immunosuppressive agents, will inevitably lead to immune disorders causing severe infectious diseases. Two cases of deep dermatophytosis with myasthenia gravis were reported before our study. Both patients underwent thymectomies and long-term oral prednisolone prior to symptom onset and had histories of superficial dermatophyte infections [12, 13]. These findings suggest that superficial dermatophyte infections cannot be ignored in patients with myasthenia gravis at potential risk for aggravation into a deep cutaneous or invasive infection.

Erythroderma is an inflammatory skin syndrome characterized by desquamation and erythema over > 90% of the body surface area. Multiple diseases with various etiopathological processes can manifest as or develop into erythroderma. Although the most common causes of erythroderma are psoriasis, eczema and drug-induced reactions, dermatophytosis may be the cause in some cases. Our patient was diagnosed with erythroderma caused by *T. rubrum* based on fungal examinations. Histological examination of the erythema confirmed the presence of a fungal infection while ruling out psoriasis, mycosis fungoides, and other potential causes. Consistent with the diagnosis of fungal infection, after treatment with antifungal agents, the erythema and scales partly subsided. To our knowledge, dermatophyte-associated erythroderma has been reported in only six patients in the English-language literature, including the current study (Table 1). Dermatophyte-induced erythroderma is rare and usually occurs in immunocompromised patients, which is consistent with Sahoo et al.’s report that underlying diseases, such as immunocompromised conditions, lymphoma, diabetes mellitus, Cushing's syndrome, and old age, are the predisposing factors causing individuals to become infected and experience heavier and more widespread fungal infections [14]. Other predisposing factors were superficial dermatophytosis and local barrier damage. Reduced skin barrier function, such as that caused by hereditary keratinization disorders, can increase the risk of serious cutaneous fungal infections [15]. *T. rubrum* can cause chronic and generalized skin infections [14]. This review revealed that *T. rubrum* was the predominant pathogenic agent. Additionally, a newly emerged fungus, *T. indotineae*, has been identified in various countries worldwide. Infections caused by *T. indotineae* often present as inflammatory, widespread, pruritic plaques on the groins, gluteal region, trunk, and face, and can occasionally lead to erythroderma [16]. Dermatophyte-associated erythroderma can be detected by direct microscopic examination and confirmed by fungal cultures and histopathological findings. Different from the cases in the literature, our patient’s pathological results showed hyphae in both the stratum corneum and dermis, suggesting possible deep dermatophytosis.

**Table 1:** Characterization of patients with dermatophyte-related erythroderma

Author, year	Age/Gender	Species/Identification	Risk factors	Location of fungi on histopathology	Treatment	Prognosis
Levene GM. 1973 [17]	36/M	*Epidermophyton floccosam.*	Chronic fungal infection, immune deficiency, lymphoma	None	GSF,3W	CR, but recurred after 3 months
Leonetti F. 1983 [18]	41/M	*T. violaceum.*	Ichthyosiform erythroderma, chronic fungal infection, DM.	ND	GSF,5 M	CR
Shelley ED. 1989 [19]	41/F	*T. rubrum.*	Lamellar ichthyosis, tinea capitis, lymphoma	Stratum corneum and hair follicles	GSF,1Y, KCZ,6 m	CR, but recurred after chemotherapy
Lyra MR. 2017 [20]	45/M	*T. tonsurans.*	Chronic alcoholic, HIV	Stratum corneum	ITR,28 d	CR
Hidayah RMN. 2021 [21]	39/M	*T. rubrum.*	Pruritic erythema, oral corticosteroid	Stratum corneum	Ketoconazole cream, urea lotion, ITR, 64d	CR
Uhrlaß S.2022 [16]	ND/M	*T. indotineae*	Long-term and extensive use of fixed-dose combination creams	ND	ND	ND
Our patient	46/M	*T. rubrum.*	Onychomycosis, tinea pedis, myasthenia gravis, thymoma, eczema, DM	Stratum corneum and dermis	Bifonazole cream, VOR(IV), ITR	Improved, but passed away due to another illness

*Abbreviations*: *M* Male, *F* Female, *DM* Diabetes mellitus, *ITR* Itraconazole, *KCZ* Ketoconazole, *GSF* Griseofulvin, *AMB* Amphotericin B, *IV* Intravenous injection, *m* Mounth, *CR* Complete response, *ND* Not described

The clinical presentation of dermatophyte-associated infections depends on many factors, including the host’s defenses against fungi, virulence of the infecting microorganism, anatomical site of infection and environmental characteristics [20]. *T. rubrum* is the most common agent of deeper dermal dermatophytosis and Majocchi’s granuloma [4]. Deeper dermal dermatophytosis differs from Majocchi’s granuloma, the former infiltrates beyond the perifollicular area and usually presents as a deeper and more severe form of infection. The lesions of deeper dermal dermatophytosis usually appear as large (> 1 cm), mostly asymptomatic, nodular lesions that are sometimes ulcerative, and superficial potassium hydroxide smears from the lesion surface are negative [22]. The lesions can occur anywhere on the body and most frequently affect the lower extremities, including the buttocks and groin [9].

Currently, few case reports exist regarding deeper dermal dermatophytosis. A review was performed of 33 previously published articles containing 50 cases of deeper dermal dermatophytosis confined to the skin and caused by *T. rubrum*, including the present case report (Table 2). Patients with deeper dermal dermatophytosis tend to be middle-aged or older adults and are more often men. The most common predisposing factor was superficial dermatophytosis, followed by receipt of immunosuppressive medications. Similar to previous reports [4, 9, 22, 23], most patients were immunosuppressed or immunocompromised, and multiple nodular lesions on the lower extremities were the most frequent clinical presentation. Kershenovich et al. [22] and Rouzaud et al. [23] found that all patients had superficial dermatophytosis lesions in association with nodules, but in our review, some patients with superficial dermatophytosis presented with only ulcers, plaques, or abscesses, not nodules. Deep dermatophytosis usually occurred shortly after initiation of immunosuppression, typically during the first year [22]. However, our patient’s infection occurred 10 years after using immunosuppressive agents; therefore, his condition may be associated with increased doses of immunosuppressive treatments in the preceding 3 months. Kershenovich et al. [22] speculated that the underlying mechanism of deep dermatophytosis was through lymphatic spread, as superficial cultures and potassium hydroxide smears of the lesions were negative. The superficial culture of the nodules on our patient was also negative, and the nodules first appeared in the lower extremities, then later spread to the trunk, upper limbs and scalp, as he had a history of onychomycosis and tinea pedis. We speculate that the *T. rubrum* first infected the lower extremities by contiguity, then spread to diffuse skin sites via vascular or lymphatic spread. In addition to our patient, another patient with *CARD9* deficiency had candidiasis. *CARD9* deficiency has been reported in chronic mucocutaneous candidiasis and cutaneous dermatophytosis. Wang et al. [4] reviewed 160 reported cases of invasive dermatophyte infections and found that six patients had concurrent *Candida* infections, and five of these patients were *CARD9*-deficient. These findings suggest that *CARD9-*deficient patients with invasive dermatophytes are prone to coinfection with *Candida* spp.

**Table 2:** Characterization of patients with deeper dermal dermatophytosis caused by *T. rubrum*

Author, year	Age/ Gender	course of disease	Underlying Disease	Immunos-uppression medicine	Superficial- mycosis	Onycho-mycosis	Clinical Presentation	Site	Histopathology Positive fungal elements	Treatment	Prognosis
Tsang,P. 1996 [24]	42/M	6w	AIDS	N	None	N	Papulonodules	Thighs	Hyphae, spores	FLU,3w	CR
Smith, 2001 [25]	34/M	ND	Acute promyelocytic leukemia	Y	None	N	Induration	Groin	Hyphae	FLU(IV,P.O), 3 m	CR
	64/F	ND	Acute myelogenous leukemia	Y	None	N	Induration	Legs	Hyphae	AMB(IV), 2w, TER, 1 m	CR
	18/M	ND	Acute lymphoblastic lymphoma	Y	None	N	Induration	Groin, lower abdomen	Hyphae	AMB(IV),2w, TER, 2w	Improved
Chastain, M. A. 2001 [26]	65/F	4 m	DM, hypertension	N	None	Y	Nodules	Forearm	Hyphae	TER, 3 m	CR
	63/M	1 m	DM and heart tx	Y	Tinea corporis and tinea pedis	N	Nodules	Lower extremities	Hyphae	TER, 2-3w	Improved
Nir-Paz, 2003 [27]	56/M	ND	Anemia, autoimmune disease	Y	None	Y	Nodules	Legs	Hyphae	ITR,3 m	CR
Tateishi, Y 2004 [28]	50/M	10 m	Atopic dermatitis	N	Tinea pedis	Y	Nodules	Diffuse	postive	ITR,4 m	Improved
Kwon, 2004 [29]	44/M	20 m	AIDS	N	None	N	Nodules	Diffuse	Hyphae	TER,21w	CR
Gong, J. Q. 2006 [30]	46/F	30y	None	N	Tinea corporis	Y	Erythema,plaques, nodules and cysts	Diffuse	Postive	ITR,5 m	CR, but recurrence
Akman, 2007 [31]	37/M	17y	Depressed cell immunity	ND	Tinea inguinalis, tinea corporis	N	Plaque	Diffuse	Hyphae, spores	GSF,3 m	CR
Lowther, 2007 [32]	64/M	2w	Asthma, rheumatoid arthritis, DM, obesity	Y	None	Y	Plaques, nodules, pustules	Hand,legs	Hyphae,spores	TER,3w	Improved, Died
Gonül, 2013 [33]	49/M	2 m	Heart tx	Y	Tinea corporis	Y	Papulonodules	Diffuse	Hyphae,spores	FLU, 2 m	CR
Matsuzaki, 2013 [12]	44/F	1y	Myasthenia gravis, DM	N	Tinea pedis	N	Tumor, nodules	Diffuse	Hyphae	ITR,4 m TER, 2y	CR
Azib, 2013 [34]	53/F	ND	Kidney tx	Y	None	Y	Nodules	Right leg, left ankle	Spores, hyphae	ND	ND
Lanternier, 2013 [35]	16/M	ND	*CARD9* mutation	N	Tinea corporis	Y	Nodules	Scalp	Hyphae	FLU, then ITR	Stable
Arunachalam, 2014 [36]	62/M	5y	Kidney tx	Y	Tinea pedis	Y	Nodule, plaques, ulcer	Left leg	Hyphae	ND	ND
Inaoki, 2015 [37]	54/M	3 m	Valvular disease of heart, nephritis and mycoplasma pneumonia	N	Tinea pedis, tinea corporis	Y	Nodules, abscess	Right lower leg	Hyphae	TER,3w	CR
Jachiet, 2015 [38]	40/M	27y	*CARD9* mutation	N	Extensive tinea corporis	Y	Plaques	Diffuse	Hyphae	ITR,TER,KCZ(ineffective) POS 8 m	CR
Kim, 2016 [39]	68/F	3 m	DM, psoriasis	Y	None	N	Nodules	Right ankle	Hyphae	ITR,3 m	CR
Su H, 2017 [40]	45/M	1y	None	N	None	Y	Plaques, nodules, ulcer	Diffuse	Hyphae	TER, 6 m	CR
Talebi-Liasi, 2017 [41]	58/M	1 m	Kidney tx	Y	None	Y	Papules, pustules	Abdomen,buttocks	Spore,	TER	ND
Kershenovich, 2017 [22]	65/M	ND	Kidney tx	Y	None	Y	Nodules	Lower limb	Hyphae, spores	TER, 1 m	CR
	41/M	ND	Kidney tx	Y	None	Y	Nodules	Neck	Hyphae, spores	TER, 6w	CR
	59/M	ND	Kidney tx	Y	None	Y	Nodules	Groin	Hyphae	FLU, 3 m	CR
	45/M	ND	Kidney tx	Y	Tinea cruris	N	Nodules	Trunk, lower limbs	Hyphae, spores	TER, 4w	CR
	69/F	ND	Colon cancer	Y	Tinea cruris	N	Nodules	Groin, pubis	Hyphae, spores	ITR,16w	CR
	61/F	ND	Lung tx	Y	None	Y	Nodules	Right shin	Hyphae, spores	TER, 8w	CR
	64/M	ND	Kidney tx	Y	None	Y	Nodules	Lower limb	Hyphae, spores GMS PAS	TER, 8w	CR
	77/M	ND	Kidney tx	Y	None	Y	Nodules	Lower limb	Hyphae, spores	FLU, 6 m	CR
	70/M	ND	Kidney tx	Y	None	Y	Nodules	Lower limb	Hyphae, spores	TER	ND
Okata-Karigane, 2018 [42]	60/M	169d	Interstitial pneumonia, polymyositis, psoriasis vulgaris	Y	None	N	Nodules, abscess	Left groin	Hyphae, spores	TER, 6 m	CR
Rouzaud, 2018 [23]	55/M	ND	Kidney tx, DM	Y	Tinea corporis (legs), Tinea cruris	Y	Nodules	Leg	Postive	POS,4 m	CR
	62/F	ND	Kidney tx, DM	Y	Tinea pedis	Y	Nodules	Diffuse	Postive	POS 12 m	CR
	57/M	ND	Kidney tx, DM	Y	Tinea corporis	N	Nodules, ulcer	Leg	Negative	TER, 8 m	CR
	71/M	ND	Kidney tx	Y	Tinea pedis	Y	Plaques, ulcer, vesicles	Toe	Postive	None	Death
	54/F	ND	Kidney tx	Y	Tinea pedis	Y	Nodules	Leg	Postive	TER 1 m	Improved Death
	55/M	ND	Kidney tx	Y	Tinea corporis	Y	Ulcer, nodule	Thigh	Postive	TER, 1 m, then VOR 0.5 m	CR
	63/M	ND	Heart tx	Y	None	Y	Ulcer, nodule	Legs, pubis	Negative	TER, 7 m	Improved
Ergen, 2018 [43]	50/M	ND	Kidney tx, diabetes	ND	None	Y	Papules	Face	Hyphae	ND	CR
Akay, 2019 [44]	65/M	1 M	Heart tx	Y	Tinea pedis	N	Nodules, ulcer	Left foot	Hyphae	ND	ND
Toussaint, 2019 [45]	52/M	10y	Inflammatory demyelinating polyneuropathy	Y	Tinea corporis	Y	Nodules, abscesses	Left hand, right arm	Hyphae	ITR, GSF, 6 m	Not improved
Bouazzi, 2019 [46]	55/F	1y	Multiple sclerosis	Y	Tinea corporis, Tinea pedis	Y	Ulcers	Lower right leg	ND	TER, 1 m	Improved
Dai, 2019 [47]	66/M	2y	Bullous pemphigoid	Y	None	N	Cysts	Lower extremities	Negative	ITR	Death
	30/M	6y	GARD9 mutation	N	Tinea corporis and tinea pedis	Y	Plaque	Left groin, scrotum	Hyphae	TER,4 m	Improved
Nazarian, 2020 [48]	31/M	15y	X-linked ichthyosis, CARD9 mutation	N	None	N	Plaques, ulcer	Diffuse	Hyphae	AMB, GSF, POS, KCZ, TER (ineffective)	Improved but recurred
Wang, 2020 [49]	48/M	19y	CARD9 mutation	N	Tinea cruris, tinea manus, pedis,	Y	Erythema, papules, nodules	Diffuse	Hyphae	TER, 3 m	CR
Sang, 2021 [50]	33/M	1y	Kidney tx	Y	Tinea cruris	N	Abscesses	Buttocks, thighs	Hyphae	TER, 3w	Death
Reis, 2021 [51]	65/M	ND	Liver tx	Y	None	N	Erythema, papules	Left buttock and thigh	Positive	ITR,1 m	Death
Our patient	46/M	1 m	Myasthenia gravis, thymoma, eczema, DM	Y	Tinea pedis	Y	Nodule, ulcer	Diffuse	Hyphae, spores	VOR(IV), ITR	Improved, but passed away due to another illness

*Abbreviations*: *M* Male, *F* Female, *ND* Not described, *y* Year, *d* Day, *tx* Transplantation, *DM* Diabetes mellitus, *Y* Yes, *N* No, *ITR* Itraconazole, *POS* Posaconazole, *VOR* Voriconazole, *TER* Terbinafine, *KCZ* Ketoconazole, *GSF* Griseofulvin, *EX* Excisional surgery, *AMB* Amphotericin B, *IV* Intravenous injection, *m* Mounth, *w* Week, *CR* Complete response, *UK* Unknown

Herein, we report the first published case of dermatophyte-induced erythroderma combined with deeper dermal dermatophytosis caused by *T. rubrum* in a patient with myasthenia gravis and diabetes. Our patient presented with an uncommon manifestation of a dermatophyte infection. Local barrier damage and systemic immunodeficiency were risk factors for the occurrence, and aggravation of these two fungal infection diseases and tinea pedis and onychomycosis may have been the origin of this infection. This case emphasizes the wide range of clinical features of dermatophytosis and suggests that for immunosuppressed patients accompanied with erythroderma and nodules with clinically unapparent manifestations, such as with our patient, clinicians should consider fungal infections. Multiple factors can lead to erythroderma, and skin biopsies, especially timely multisite biopsies, can help determine the underlying causes. Dermatologists should also be aware that patients presenting with skin tinea and immunocompromised diseases tend to have a higher risk of developing invasive dermatophytic infections or disseminated fungal infections. Thus, microscopic fungal examinations and fungal cultures should be performed for these patients, and superficial dermatophytosis must be treated promptly, before immunosuppression occurs. Dermatologists should continue to closely observe these patients.

## Data Availability

The datasets generated and/or analysed during the current study are available in the [ScienceDB] repository, (https://www.scidb.cn/detail?dataSetId=78a315bc09524b55bffc8c158eeb865a; CSTR: 31253.11.sciencedb.11620).

## Abbreviations

*T. rubrum*: *Trichophyton rubrum*
HIV: Human immunodeficiency virus
HE: Hematoxylin and eosin
PAS: Periodic acid-Schiff
